# A five-coordinate cobalt bis­(di­thiol­ene)–phosphine complex [Co(pdt)_2_(PTA)] (pdt = phenyl­dithiol­ene; PTA = 1,3,5-tri­aza-7-phosphaadamantane)

**DOI:** 10.1107/S2056989020005447

**Published:** 2020-04-24

**Authors:** DaShawn Williams, Jacob P. Brannon, Perumalreddy Chandrasekaran, S. Chantal E. Stieber

**Affiliations:** a Department of Chemistry & Biochemistry, Lamar University, 4400 S. M.L.K. King Jr. Pkwy, Beaumont, TX 77705, USA; b Department of Chemistry & Biochemistry, California State Polytechnic University, Pomona, 3801 W. Temple Ave., Pomona, CA 91768, USA

**Keywords:** cobalt complex, di­thiol­ene, redox-active, non-innocent ligand, PTA ligand, crystal structure

## Abstract

The title compound, bis­(1,2-diphenyl-2-sulfanyl­idene­ethane­thiol­ato-κ^2^ *S*,*S*′)(1,3,5-tri­aza-7-phosphaadamantane-κ*P*)cobalt(II) dichloromethane hemisolvate, [Co(pdt)_2_(PTA)]·0.5C_2_H_4_Cl_2_ or [Co(C_14_H_10_S_2_)_2_(C_6_H_12_N_3_P)]·0.5C_2_H_4_Cl_2_, contains two phenyl­dithiol­ene (pdt) ligands and a 1,3,5-tri­aza-7-phosphaadamantane (PTA) ligand bound to cobalt with the solvent 1,2-di­chloro­ethane mol­ecule located on an inversion center. The cobalt core exhibits an approximately square-pyramidal geometry with partially reduced thienyl radical monoanionic ligands. The supra­molecular network is consolidated by hydrogen-bonding inter­actions primarily with nitro­gen, sulfur and chlorine atoms, as well as parallel displaced π-stacking of the aryl rings. The UV–vis, IR, and CV data are also consistent with monoanionic di­thiol­ene ligands and an overall Co^II^ oxidation state.

## Abstract 2

The synthesis and crystal structure are reported for a five-coordinate cobalt di­thiol­ene-phosphine complex [Co(pdt)_2_(PTA)] (pdt = phenyl­dithiol­ene; S_2_C_2_Ph_2_), produced by PTA ligand-induced cleavage of the cobalt bis­(di­thiol­ene) dimer [Co_2_(pdt)_4_].

## Chemical context   

Transition-metal complexes of 1,3,5-tri­aza-7-phosphaadamantane (PTA) and related ligands have attracted much attention because of their potential as water-soluble catalysts, materials, and therapeutic agents (Guerriero *et al.*, 2018 ▸). The small cone angle (103°) of the PTA ligand combined with the high thermal and chemical stability, and high hydro­philicity makes it unique among phosphine ligands (Phillips *et al.*, 2004 ▸). Electronically, the PTA ligand is much less electron donating than PMe_3_, while a slightly better electron donor than PPh_3_ (Darensbourg *et al.*, 1999 ▸). However, the formation of heteroleptic di­thiol­ene-phosphine complexes from the corresponding homoleptic metal-di­thiol­ene has not been fully explored (Natarajan *et al.*, 2017 ▸). Reactions of homoleptic metal-di­thiol­enes with phosphines to produce heteroleptic complexes have exhibited inter­esting metal–ligand redox inter­play as a result of the redox-active or non-innocent nature of di­thiol­ene ligands (Chandrasekaran *et al.*, 2014 ▸). In this context, phosphine-induced cleavage of the iron and cobalt bis­(di­thiol­ene) dimer to yield five-coordinate bis­(di­thiol­ene)phosphine has been explored in depth with PPh_3_ and PMe_3_ ligands (Selby-Karney *et al.*, 2017 ▸; Yu *et al.*, 2007 ▸). These complexes were all synthesized from the corresponding bis(di­thiol­ene) metal dimer complexes followed by addition of an excess of phosphine ligand to form bis­(di­thiol­ene) metal complexes bound to an additional phosphine ligand. The resulting [*M*(adt)_2_(P*R*_3_)] (*M* = Co, Fe; adt = *para*-anisyl­phenyl­dithiol­ene­; P*R*_3_ = PMe_3_ or PPh_3_) complexes have approximately square-pyramidal geometries at the metal center.
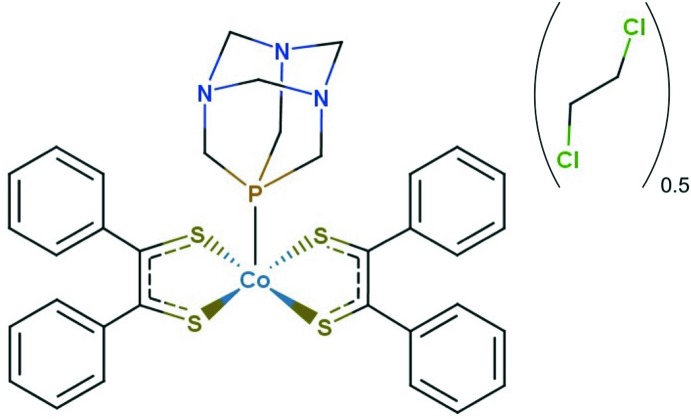


Herein, we report the synthesis and crystal structure of a five-coordinate cobalt di­thiol­ene-phosphine complex [Co(pdt)_2_(PTA)] (pdt = phenyl­dithiol­ene, S_2_C_2_Ph_2_), produced by PTA ligand-induced cleavage of the cobalt bis­(di­thiol­ene) dimer [Co_2_(pdt)_4_].

## Structural commentary   

**Figure 1:**
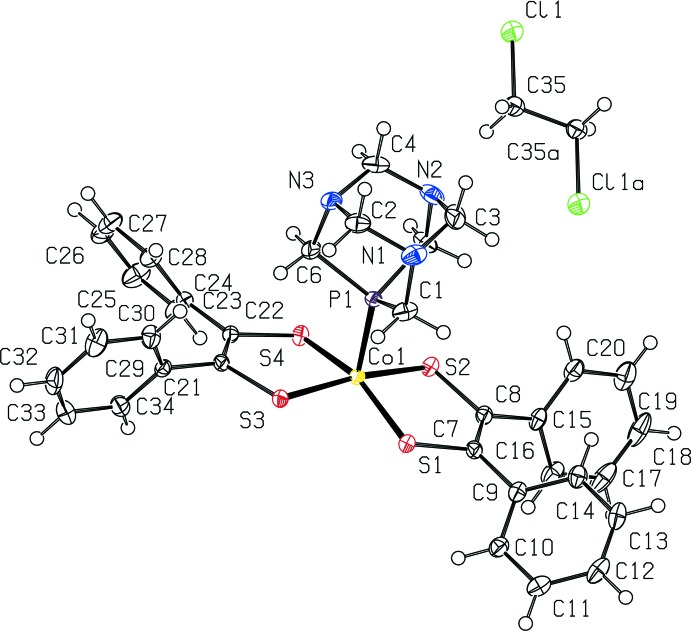
View of [Co(C_14_H_10_S_2_)_2_(C_6_H_12_N_3_P)]·0.5C_2_H_4_Cl_2_ with 50% probability ellipsoids.

**Figure 2:**
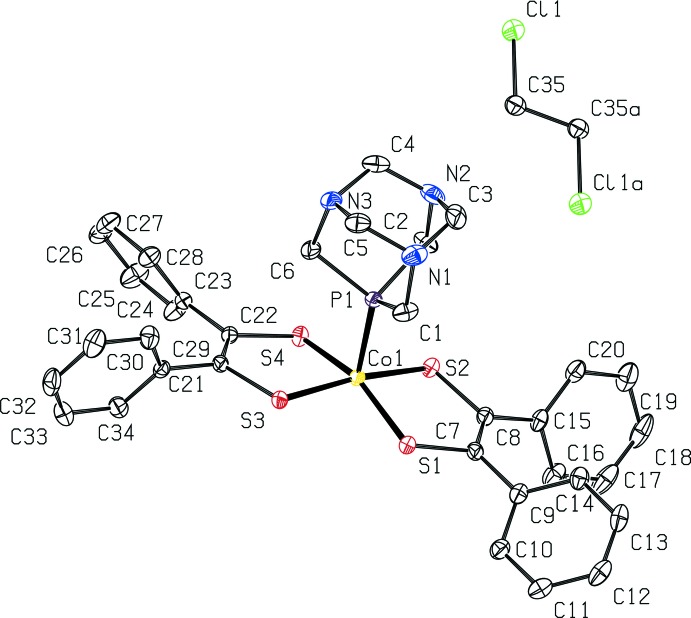
View of [Co(C_14_H_10_S_2_)_2_(C_6_H_12_N_3_P)]·0.5C_2_H_4_Cl_2_ with 50% probability ellipsoids. H atoms omitted for clarity.

[Co(pdt)_2_(PTA)] co-crystallizes with one mol­ecule of 1,2-di­chloro­ethane where half of the solvent mol­ecule is symmetry generated, as shown in Fig. 1 ▸. The structure without hydrogen atoms is depicted in Fig. 2 ▸ for clarity. Each di­thiol­ene ligand coordinates to the cobalt center in a κ^2^ fashion *via* the sulfur atoms, and PTA coordinates *via* the apical phospho­rous atom. The cobalt di­thiol­ene core is approximately planar, with angles of 89.73 (2)° for S1—Co1—S2, 88.93 (2)° for S1—Co1—S3, 88.41 (2)° for S2—Co1—S4, and 89.90 (2)° for S3—Co1—S4. The sum of the angles is 356.97 (4)°, consistent with only a slight distortion from planarity. The PTA ligand occupies a 5^th^ coordination site with angles of 101.94 (2)° for P1—Co1—S1, 98.12 (2)° for P1—Co1—S2, 90.97 (2)° for P1—Co1—S3, and 97.22 (2)° for P1–Co1—S4. Therefore, the overall geometry of [Co(pdt)_2_(PTA)] is approximately square pyramidal.

The distances from the cobalt atom to the sulfur ligands are 2.1620 (5) Å for Co1—S1, 2.1669 (6) Å for Co1—S2, 2.1685 (5) Å for Co1—S3, and 2.1487 (5) Å for Co1—S4. These are mostly within the range of Co—S distances of 2.1659 (9)–2.1765 (9) Å reported for the *p*-anisyl-substituted analogues with PMe_3_ and PPh_3_ (Selby-Karney *et al.*, 2017 ▸; Yu *et al.*, 2007 ▸). The Co1—P1 distance is 2.1424 (5) Å, which is shorter than the distances of 2.163 (1) and 2.192 (1) Å reported for the *p*-anisyl-substituted analogues with PMe_3_ and PPh_3_, respectively (Selby-Karney *et al.*, 2017 ▸; Yu *et al.*, 2007 ▸). The decreasing length of the Co1—P1 bond for PPh_3_ > PMe_3_ > PTA is not consistent with the σ-donating ability of the phosphine which increases from PPh_3_ < PTA < PMe_3_. Instead, the short Co1—P1 bond for [Co(pdt)_2_(PTA)] is attributed to the small cone angle of 103° (Phillips *et al.*, 2004 ▸) as compared to the cone angle of 118° for PMe_3_ and 145° for PPh_3_ (Bilbrey *et al.*, 2013 ▸).

The sulfur–carbon distances are consistent with a partially reduced ligand thienyl radical monoanion with distances of 1.728 (2) Å for S1—C7, 1.719 (2) Å for S2—C8, 1.729 (2) Å for S3—C21, and 1.731 (2) Å for S4—C22. These are mostly within the range of S—C distances of 1.721 (2)–1.726 (3) and 1.730 (3)–1.742 (3) Å reported for the *p*-anisyl-substituted analogues with PMe_3_ and PPh_3_, respectively (Selby-Karney *et al.*, 2017 ▸; Yu *et al.*, 2007 ▸). The C7—C8 distance of 1.372 (3) Å and the C21—C22 distance of 1.365 (3) Å are also consistent with this description.

## Supra­molecular features   

**Figure 3:**
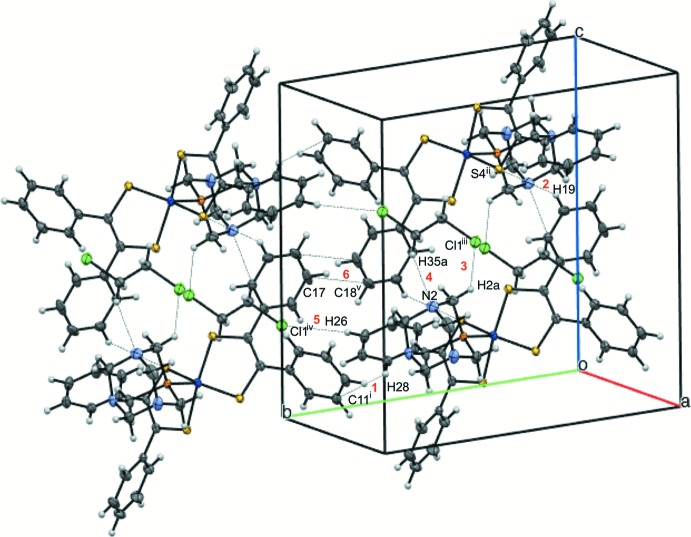
View of two mol­ecules of [Co(C_14_H_10_S_2_)_2_(C_6_H_12_N_3_P)]·0.5C_2_H_4_Cl_2_ within the unit cell and one additional translation of the unit cell along *b*, at 50% probability ellipsoids. Close contacts, including hydrogen bonds are labeled 1–6 with distances given in Table 1 ▸.

Two mol­ecules of [Co(pdt)_2_(PTA)] and one mol­ecule of 1,2-dicholorethane are present in the unit cell, as depicted in Fig. 3 ▸. The two metal complexes in the unit cell are related by an inversion operation with the inversion center on the 1,2-di­chloro­ethane and the cobalt di­thiol­ene cores being approximately parallel to each other. Six close contacts within the supra­molecular framework were identified (Fig. 3 ▸, Table 1 ▸), resulting primarily from hydrogen-bonding inter­actions with nitro­gen, sulfur and chlorine. Hydrogen bonds with sulfur and nitro­gen include the C19—H19⋯S4^ii^ distance of 2.78 Å (Figs. 3 ▸, #2 ▸) and the C35—H35*a*⋯N2 distance of 2.69 Å (Figs. 3 ▸, #4 ▸). Weaker hydrogen bonds with chlorine include the C2—H2*a*⋯Cl1^iii^ distance of 2.88 Å (Figs. 3 ▸, #3 ▸) and the C26—H26⋯Cl1^iv^ distance of 2.95 Å (Figs. 3 ▸, #5). Close contacts with carbon and hydrogen atoms include a C28—H28⋯C11^i^ distance of 2.83 Å (Figs. 3 ▸, #1 ▸; see Table 1 ▸ for symmetry operators).

**Figure 4:**
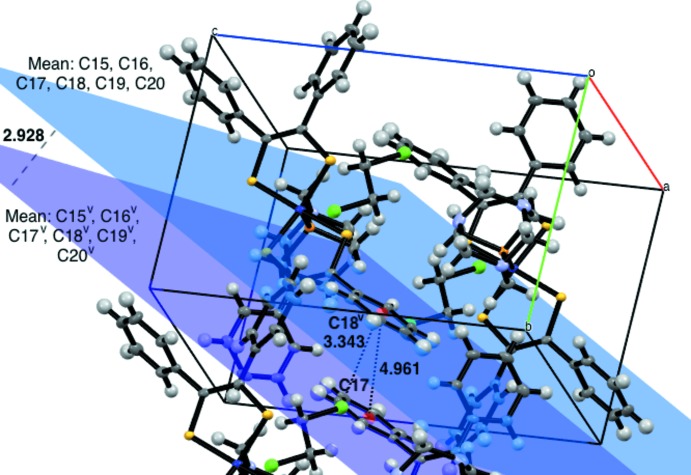
View of two mol­ecules of [Co(C_14_H_10_S_2_)_2_(C_6_H_12_N_3_P)]·0.5C_2_H_4_Cl_2_ within the unit cell and one additional translation of the unit cell along *b*, at 50% probability ellipsoids. Planes defined by aryl rings containing C15–C20 along with the corresponding centroids are depicted to highlight parallel displaced π-stacking of aryl rings.

When the unit cell is grown along the *b* axis, parallel displaced π-stacking of the aryl rings is revealed (Fig. 4 ▸). Planes defined by atoms C15–C20 (Fig. 4 ▸, blue) and C15^v^–C20^v^ [Fig. 4 ▸, purple; symmetry code: (v) 1 − *x*, −*y*, 1 − *z*] within the unit cell are parallel, with a distance of 2.928 Å between planes and a distance of 4.961 Å between the respective centroids defined by the same atoms. The shortest atomic distance is between the carbon atoms of the aryl rings between unit cells with C17⋯C18^v^ being 3.343 (3) Å apart (Figs. 3 ▸, #6; Fig. 4 ▸).

## Database survey   

A survey of the Cambridge Structural Database (Web accessed March 26, 2020; Groom *et al.*, 2016 ▸) and SciFinder (SciFinder, 2020 ▸) yielded no exact matches for reported structures of this complex. Structures with two di­thiol­ene ligands with *p*-anisyl substitution bound to Co and an additional coordinated phosphine ligand were reported with PMe_3_ coordination (Selby-Karney *et al.*, 2017 ▸), and PPh_3_ coordination (Yu *et al.*, 2007 ▸). Both reported complexes also have approximately square pyramidal geometry at the cobalt center with slight deviations. The PPh_3_ complex exhibits the largest distortion from planarity with a sum of angles around cobalt of 353.89 (6)°, while the sum of the angles is 356.97 (6)° for the PMe_3_ complex. Similarly, the phosphine in PPh_3_ is axially distorted because of the steric bulk of the phenyl groups, resulting in two more obtuse bond angles for S2—Co1—P1 and S3—Co1—P1 of 101.31 (3) and 106.6 (3)°, respectively. The other bond angles of 92.81 (3)° for S1—Co1–P1 and 97.13 (3)° for S4—Co1—P1 are within the range of S—Co1—P1 angles of 91.19 (3) to 99.65 (3)° observed for the PMe_3_ complex.

## Spectroscopic analysis   

**Figure 5:**
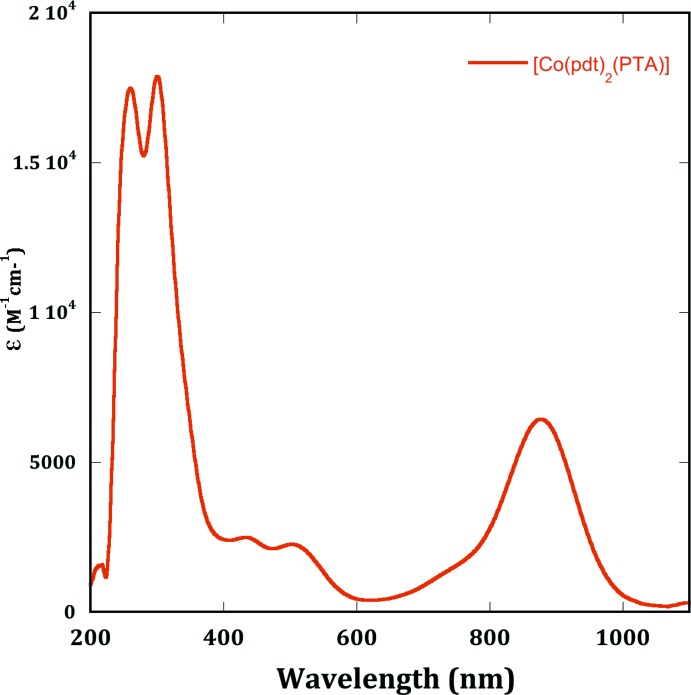
UV–vis spectrum of [Co(pdt)_2_(PTA)] in di­chloro­methane solution at 25°C.

The UV–vis characterization of [Co(pdt)_2_(PTA)] was conducted in di­chloro­methane (Fig. 5 ▸) and revealed a strong absorption at 877 nm with a molar absorptivitiy of 6428 M^−1^cm^−1^. In the related *p*-anisyl-substituted cobalt complex bound to PMe_3_ a similar absorption was observed at 905 nm. This is attributed to a ligand-to-ligand charge-transfer (LLCT) transition, based on comparison with the related iron complex with PPh_3_ (Yu *et al.*, 2007 ▸) and related di­thiol­ene metal complexes (Ray *et al.*, 2005 ▸). In the iron PPh_3_ complex, the absorption occurred at 720 nm and disappeared upon conversion to the homoleptic iron bis­(di­thiol­ene) complex. The IR signal for [Co(pdt)_2_(PTA)] at 1157.61 cm^−1^ is characteristic of monoanionic di­thiol­enes with a π-radical when coordinated to metals, and is attributed to ν(C=S·) (Patra *et al.*, 2006 ▸). Combined, the IR and UV–vis characterization are consistent with two monoanionic di­thiol­ene ligands bound to a Co^II^ center.

## Electrochemical analysis   

**Figure 6:**
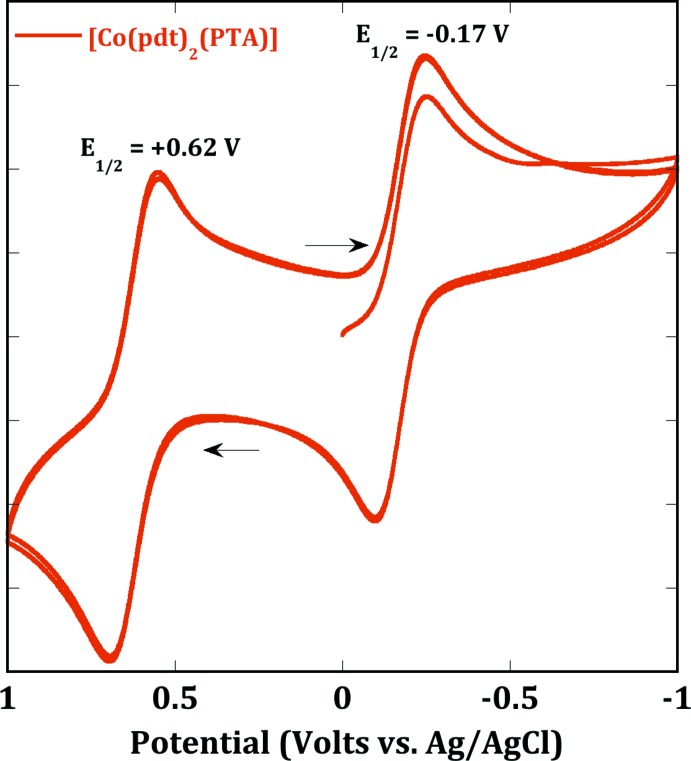
Cyclic voltammetry of [Co(pdt)_2_(PTA)] in CH_2_Cl_2_ recorded using a platinum working electrode and [^*n*^Bu_4_N][PF_6_] as electrolyte with a scan rate of 100 mV s^−1^ at 25°C.

**Figure 7:**
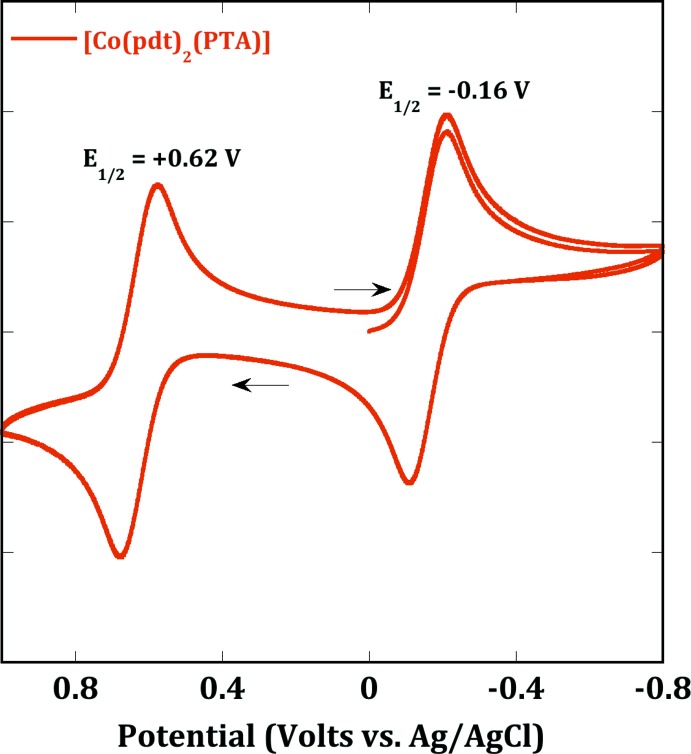
Cyclic voltammetry of [Co(pdt)_2_(PTA)] in CH_2_Cl_2_ recorded using a glassy carbon working electrode and [^*n*^Bu_4_N][PF_6_] as electrolyte with a scan rate of 100 mV s^−1^ at 25°C.

The cyclic voltammogram (CV) of [Co(pdt)_2_(PTA)] was collected in a solution of dichoro­methane with a platinum working electrode (Fig. 6 ▸) and a glassy carbon working electrode (Fig. 7 ▸). Both CVs display two reversible waves with the first one at *E*_1/2_ = +0.62 with both electrodes, and a second one at *E*_1/2_ = −0.17 V with the platinum electrode and *E*_1/2_ = −0.16 V with the glassy carbon electrode. The reversible oxidation wave at +0.62 V is attributed to a metal-centered redox event. The second oxidation at −0.17 V is attributed to ligand oxidation, by comparison to other metal di­thiol­ene complexes (Patra *et al.*, 2006 ▸).

## Synthesis and crystallization   

A 50 mL Schlenk flask containing a stir bar was charged with [Co_2_(pdt)_4_] (0.300 g, 0.275 mmol) and PTA (0.144 g; 0.551 mmol) under an N_2_ atmosphere. To this mixture of solids, 20 mL of CH_2_Cl_2_ were added and stirred for 4 h at room temperature. The solvent was removed under reduced pressure and the resulting dark-orange solid was washed with 3 × 5 mL of Et_2_O and dried under vacuum. The product was stable under reduced pressure and at room temperature. Yield: 92% (0.357 g, 0.509 mmol). Crystals suitable for X-ray diffraction were grown by the vapor diffusion method with diffusion of pentane over a 1,2-di­chloro­ethane solution of the compound. UV–Vis spectra were obtained at ambient temperature with a Varian Cary 50 diode array spectrometer, while IR spectra were taken neat with an ALPHA FTIR instrument. Electrochemical measurements were performed with a CHI600E electroanalyzer workstation using an Ag/AgCl reference electrode, a platinum disk working electrode, a platinum wire auxiliary electrode, and [^*n*^Bu_4_N][PF_6_] as the supporting electrolyte in CH_2_Cl_2_. Under these conditions, the [Cp_2_Fe]^+^/ Cp_2_Fe couple consistently occurred at +440 mV. UV–vis in CH_2_Cl_2_: [λ_max_, nm (∊_M_, M^−1^cm^−1^)]: 301 nm (17881), 877 nm (6428). IR spectroscopy (cm^−1^): 3366.53 (*w*), 3054.34 (*w*), 2928.02 (*w*), 2869.68 (*w*), 1592.50 (*w*), 1440.63 (*m*), 1415.07 (*s*), 1275.25 (*m*), 1240.54 (*w*), 1157.61 (*m*), 1091.56 (*s*), 1012.49 (*m*), 969.10 (*s*), 940.65 (*vs*), 739.50 (*s*), 693.31 (*s*).

## Refinement   

Crystal data, data collection and structure refinement details are summarized in Table 2 ▸. Hydrogen atoms were placed in calculated positions with C—H distances of 0.95 and 0.99 Å for CH and CH_2_, respectively, and refined using a riding model with *U*_iso_(H) = 1.2 *U*_eq_(C) for CH and CH_2_.

## Supplementary Material

Crystal structure: contains datablock(s) I. DOI: 10.1107/S2056989020005447/zl2776sup1.cif

Structure factors: contains datablock(s) I. DOI: 10.1107/S2056989020005447/zl2776Isup2.hkl

CCDC reference: 1986931

Additional supporting information:  crystallographic information; 3D view; checkCIF report

## Figures and Tables

**Table 1:** Hydrogen-bond geometry (Å, °)

*D*—H⋯*A*	*D*—H	H⋯*A*	*D*⋯*A*	*D*—H⋯*A*
C28—H28⋯C11^i^	0.95	2.83	3.575 (3)	136
C19—H19⋯S4^ii^	0.95	2.78	3.513 (3)	135
C2—H2a⋯Cl1^iii^	0.99	2.88	3.582 (2)	129
C35—H35a⋯N2	0.99	2.69	3.297 (3)	120
C26—H26⋯Cl1^iv^	0.95	2.95	3.824 (2)	154

Symmetry codes: (i) 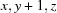; (ii) 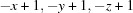; (iii) 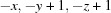; (iv) 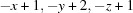.

**Table 2:** Experimental details

Crystal data	
Chemical formula	[Co(C_14_H_10_S_2_)_2_(C_6_H_12_N_3_P)·0.5C_2_H_4_Cl_2_
*M*_r_	750.24
Crystal system, space group	Triclinic, *P* 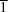
Temperature (K)	113
*a*, *b*, *c* (Å)	9.0954 (7), 13.4319 (9), 14.7905 (10)
α, β, γ (°)	97.074 (3), 94.680 (3), 107.354 (3)
*V* (Å^3^)	1698.1 (2)
*Z*	2
Radiation type	Mo *K*α
μ (mm^−1^)	0.91
Crystal size (mm)	0.57 × 0.32 × 0.14

Data collection	
Diffractometer	Bruker D8 Venture Kappa
Absorption correction	Multi-scan (*SADABS*; Krause *et al.*, 2015 ▸)
*T*_min_, *T*_max_	0.661, 0.746
No. of measured, independent and observed [*I* > 2σ(*I*)] reflections	46909, 7487, 6778
*R*_int_	0.045
(sin θ/λ)_max_ (Å^−1^)	0.642

Refinement	
*R*[*F* ^2^ > 2σ(*F* ^2^)], *wR*(*F* ^2^), *S*	0.031, 0.083, 1.06
No. of reflections	7487
No. of parameters	406
H-atom treatment	H-atom parameters constrained
Δρ_max_, Δρ_min_ (e Å^−3^)	0.73, −0.40

Computer programs: *APEX3* and *SAINT* (Bruker, 2017 ▸), *SHELXT2014* (Sheldrick, 2015*a* ▸), *SHELXL2017* (Sheldrick, 2015*b* ▸), *OLEX2* (Dolomanov *et al.*, 2009 ▸), *Mercury* (Macrae *et al.*, 2020 ▸) and *publCIF* (Westrip, 2010 ▸).

## supplementary crystallographic information

### Crystal data

**Table:**

[Co(C_14_H_10_S_2_)_2_(C_6_H_12_N_3_P)·0.5C_2_H_4_Cl_2_	*Z* = 2
*M**_r_* = 750.24	*F*(000) = 776
Triclinic, *P*1	*D*_x_ = 1.467 Mg m^−^^3^
*a* = 9.0954 (7) Å	Mo *K*α radiation, λ = 0.71073 Å
*b* = 13.4319 (9) Å	Cell parameters from 9901 reflections
*c* = 14.7905 (10) Å	θ = 3.5–34.9°
α = 97.074 (3)°	µ = 0.91 mm^−^^1^
β = 94.680 (3)°	*T* = 113 K
γ = 107.354 (3)°	Prism, black
*V* = 1698.1 (2) Å^3^	0.57 × 0.32 × 0.14 mm

### Data collection

**Table:**

Bruker D8 Venture Kappa diffractometer	6778 reflections with *I* > 2σ(*I*)
Radiation source: microfocus sealed tube	*R*_int_ = 0.045
φ and ω scans	θ_max_ = 27.1°, θ_min_ = 2.6°
Absorption correction: multi-scan (SADABS; Krause *et al.*, 2015)	*h* = −11→11
*T*_min_ = 0.661, *T*_max_ = 0.746	*k* = −17→17
46909 measured reflections	*l* = −18→18
7487 independent reflections	

### Refinement

**Table:**

Refinement on *F*^2^	Primary atom site location: structure-invariant direct methods
Least-squares matrix: full	Secondary atom site location: difference Fourier map
*R*[*F*^2^ > 2σ(*F*^2^)] = 0.031	Hydrogen site location: mixed
*wR*(*F*^2^) = 0.083	H-atom parameters constrained
*S* = 1.06	*w* = 1/[σ^2^(*F*_o_^2^) + (0.0327*P*)^2^ + 1.8472*P*] where *P* = (*F*_o_^2^ + 2*F*_c_^2^)/3
7487 reflections	(Δ/σ)_max_ = 0.002
406 parameters	Δρ_max_ = 0.73 e Å^−^^3^
0 restraints	Δρ_min_ = −0.40 e Å^−^^3^

### Special details

**Table:**

Geometry. All esds (except the esd in the dihedral angle between two l.s. planes) are estimated using the full covariance matrix. The cell esds are taken into account individually in the estimation of esds in distances, angles and torsion angles; correlations between esds in cell parameters are only used when they are defined by crystal symmetry. An approximate (isotropic) treatment of cell esds is used for estimating esds involving l.s. planes.

### Fractional atomic coordinates and isotropic or equivalent isotropic displacement parameters (Å^2^)

**Table:**

	*x*	*y*	*z*	*U*_iso_*/*U*_eq_	
C1	0.2841 (2)	0.44175 (16)	0.13148 (14)	0.0238 (4)	
H1A	0.327954	0.444029	0.072381	0.029*	
H1B	0.262644	0.369103	0.145884	0.029*	
Co1	0.64640 (3)	0.50794 (2)	0.24515 (2)	0.01404 (7)	
N1	0.1384 (2)	0.46794 (14)	0.12245 (13)	0.0265 (4)	
Cl1	−0.03998 (7)	0.64232 (4)	0.56103 (4)	0.03132 (12)	
S1	0.59835 (5)	0.34986 (3)	0.17196 (3)	0.01709 (10)	
P1	0.42620 (5)	0.53418 (4)	0.22219 (3)	0.01466 (10)	
C2	0.3048 (2)	0.52908 (19)	0.31626 (14)	0.0262 (4)	
H2A	0.284600	0.459895	0.337846	0.031*	
H2B	0.360251	0.584927	0.368404	0.031*	
S2	0.61456 (5)	0.44452 (3)	0.37252 (3)	0.01757 (10)	
N2	0.1566 (2)	0.54440 (16)	0.28436 (13)	0.0279 (4)	
C3	0.0688 (2)	0.46267 (18)	0.20808 (17)	0.0319 (5)	
H3A	0.058232	0.392641	0.226358	0.038*	
H3B	−0.036915	0.468504	0.196775	0.038*	
S3	0.71513 (5)	0.56935 (3)	0.12089 (3)	0.01568 (10)	
N3	0.25473 (19)	0.65989 (13)	0.17147 (12)	0.0225 (3)	
C4	0.1799 (2)	0.64750 (18)	0.25548 (15)	0.0276 (4)	
H4A	0.244268	0.702669	0.305799	0.033*	
H4B	0.077805	0.659288	0.245456	0.033*	
S4	0.76992 (5)	0.65936 (3)	0.32479 (3)	0.01744 (10)	
C20	0.4388 (3)	0.24991 (15)	0.47931 (13)	0.0238 (4)	
H20	0.373511	0.292452	0.470978	0.029*	
C21	0.8023 (2)	0.70357 (14)	0.15445 (13)	0.0156 (3)	
C22	0.8262 (2)	0.74384 (14)	0.24571 (12)	0.0150 (3)	
C23	0.8953 (2)	0.85744 (14)	0.28453 (13)	0.0169 (3)	
C24	1.0120 (2)	0.88975 (16)	0.35854 (15)	0.0265 (4)	
H24	1.051162	0.839134	0.382873	0.032*	
C25	1.0716 (3)	0.99529 (17)	0.39707 (17)	0.0330 (5)	
H25	1.151905	1.016440	0.447350	0.040*	
C26	1.0157 (3)	1.06997 (16)	0.36321 (16)	0.0291 (5)	
H26	1.057262	1.142228	0.389814	0.035*	
C27	0.8984 (3)	1.03871 (16)	0.29017 (15)	0.0279 (4)	
H27	0.858604	1.089600	0.266868	0.033*	
C28	0.8388 (2)	0.93339 (16)	0.25086 (14)	0.0228 (4)	
H28	0.758727	0.912696	0.200497	0.027*	
C29	0.8531 (2)	0.76591 (14)	0.07994 (12)	0.0159 (3)	
C30	0.7460 (2)	0.78816 (16)	0.01894 (14)	0.0229 (4)	
H30	0.638499	0.763550	0.025136	0.027*	
C31	0.7949 (3)	0.84572 (17)	−0.05041 (14)	0.0293 (5)	
H31	0.721373	0.861822	−0.090688	0.035*	
C32	0.9510 (3)	0.87993 (17)	−0.06125 (15)	0.0300 (5)	
H32	0.984506	0.918433	−0.109521	0.036*	
C33	1.0578 (3)	0.85777 (18)	−0.00151 (16)	0.0311 (5)	
H33	1.164953	0.881375	−0.008711	0.037*	
C34	1.0094 (2)	0.80118 (16)	0.06902 (14)	0.0240 (4)	
H34	1.083649	0.786567	0.109948	0.029*	
C35	0.0253 (2)	0.55372 (15)	0.48550 (14)	0.0226 (4)	
H35A	−0.019761	0.549633	0.421409	0.027*	
H35B	0.139845	0.579612	0.488730	0.027*	
C5	0.1638 (2)	0.57416 (17)	0.09845 (14)	0.0252 (4)	
H5A	0.217909	0.579321	0.042966	0.030*	
H5B	0.061620	0.584021	0.082845	0.030*	
C8	0.5691 (2)	0.30997 (14)	0.34348 (13)	0.0173 (4)	
C7	0.5610 (2)	0.26674 (14)	0.25345 (13)	0.0173 (4)	
C6	0.4165 (2)	0.65983 (16)	0.18756 (15)	0.0232 (4)	
H6A	0.474959	0.719078	0.236339	0.028*	
H6B	0.465750	0.670512	0.130753	0.028*	
C9	0.5189 (2)	0.15214 (14)	0.21930 (13)	0.0183 (4)	
C19	0.4218 (3)	0.18900 (17)	0.54991 (15)	0.0341 (5)	
H19	0.342685	0.188436	0.588150	0.041*	
C10	0.5971 (2)	0.11500 (15)	0.15203 (14)	0.0215 (4)	
H10	0.680174	0.163381	0.129720	0.026*	
C11	0.5548 (3)	0.00803 (16)	0.11733 (14)	0.0252 (4)	
H11	0.608667	−0.016269	0.071427	0.030*	
C12	0.4341 (3)	−0.06324 (16)	0.14966 (15)	0.0273 (4)	
H12	0.405635	−0.136409	0.126284	0.033*	
C14	0.3962 (2)	0.07902 (15)	0.25086 (13)	0.0216 (4)	
H14	0.340984	0.102673	0.296318	0.026*	
C13	0.3552 (2)	−0.02763 (16)	0.21590 (14)	0.0260 (4)	
H13	0.272179	−0.076569	0.237726	0.031*	
C15	0.5509 (2)	0.24862 (14)	0.42110 (13)	0.0190 (4)	
C16	0.6491 (2)	0.18828 (16)	0.43625 (15)	0.0268 (4)	
H16	0.726843	0.187116	0.397345	0.032*	
C17	0.6331 (3)	0.12979 (17)	0.50830 (17)	0.0356 (6)	
H17	0.701147	0.089642	0.518871	0.043*	
C18	0.5191 (3)	0.12967 (17)	0.56452 (15)	0.0367 (6)	
H18	0.507881	0.088833	0.613074	0.044*	

### Atomic displacement parameters (Å^2^)

**Table:**

	*U*^11^	*U*^22^	*U*^33^	*U*^12^	*U*^13^	*U*^23^
C1	0.0212 (10)	0.0219 (10)	0.0265 (10)	0.0092 (8)	−0.0051 (8)	−0.0047 (8)
Co1	0.01191 (12)	0.01288 (12)	0.01779 (13)	0.00396 (9)	0.00186 (9)	0.00394 (9)
N1	0.0189 (8)	0.0249 (9)	0.0342 (10)	0.0091 (7)	−0.0055 (7)	−0.0012 (7)
Cl1	0.0363 (3)	0.0239 (2)	0.0365 (3)	0.0108 (2)	0.0142 (2)	0.0052 (2)
S1	0.0180 (2)	0.0140 (2)	0.0196 (2)	0.00467 (17)	0.00375 (16)	0.00368 (16)
P1	0.0124 (2)	0.0152 (2)	0.0172 (2)	0.00534 (17)	0.00227 (16)	0.00254 (17)
C2	0.0196 (10)	0.0406 (12)	0.0239 (10)	0.0142 (9)	0.0075 (8)	0.0103 (9)
S2	0.0199 (2)	0.0142 (2)	0.0185 (2)	0.00479 (17)	0.00112 (16)	0.00396 (16)
N2	0.0189 (8)	0.0421 (11)	0.0296 (9)	0.0159 (8)	0.0089 (7)	0.0117 (8)
C3	0.0133 (9)	0.0310 (11)	0.0524 (14)	0.0047 (8)	0.0036 (9)	0.0160 (10)
S3	0.0155 (2)	0.0135 (2)	0.0172 (2)	0.00298 (16)	0.00296 (16)	0.00246 (16)
N3	0.0173 (8)	0.0213 (8)	0.0319 (9)	0.0101 (7)	0.0027 (7)	0.0054 (7)
C4	0.0239 (10)	0.0323 (11)	0.0301 (11)	0.0168 (9)	0.0034 (8)	−0.0021 (9)
S4	0.0192 (2)	0.0150 (2)	0.0166 (2)	0.00298 (17)	0.00080 (16)	0.00396 (16)
C20	0.0338 (11)	0.0164 (9)	0.0207 (9)	0.0071 (8)	0.0032 (8)	0.0027 (7)
C21	0.0110 (8)	0.0146 (8)	0.0216 (9)	0.0036 (6)	0.0026 (6)	0.0051 (7)
C22	0.0114 (8)	0.0152 (8)	0.0195 (8)	0.0049 (7)	0.0010 (6)	0.0062 (7)
C23	0.0160 (8)	0.0154 (8)	0.0196 (9)	0.0047 (7)	0.0046 (7)	0.0031 (7)
C24	0.0269 (11)	0.0174 (9)	0.0332 (11)	0.0077 (8)	−0.0063 (8)	0.0012 (8)
C25	0.0308 (12)	0.0231 (11)	0.0394 (12)	0.0082 (9)	−0.0120 (9)	−0.0053 (9)
C26	0.0332 (12)	0.0160 (9)	0.0352 (12)	0.0065 (8)	0.0022 (9)	−0.0030 (8)
C27	0.0390 (12)	0.0203 (10)	0.0285 (10)	0.0154 (9)	0.0035 (9)	0.0043 (8)
C28	0.0264 (10)	0.0210 (9)	0.0216 (9)	0.0093 (8)	−0.0003 (8)	0.0028 (7)
C29	0.0183 (9)	0.0123 (8)	0.0166 (8)	0.0043 (7)	0.0022 (7)	0.0016 (6)
C30	0.0187 (9)	0.0226 (10)	0.0248 (10)	0.0028 (8)	−0.0016 (7)	0.0063 (8)
C31	0.0363 (12)	0.0249 (10)	0.0236 (10)	0.0059 (9)	−0.0063 (9)	0.0072 (8)
C32	0.0417 (13)	0.0224 (10)	0.0240 (10)	0.0036 (9)	0.0089 (9)	0.0099 (8)
C33	0.0280 (11)	0.0315 (11)	0.0359 (12)	0.0067 (9)	0.0150 (9)	0.0125 (9)
C34	0.0205 (10)	0.0249 (10)	0.0288 (10)	0.0076 (8)	0.0063 (8)	0.0091 (8)
C35	0.0228 (10)	0.0240 (10)	0.0227 (9)	0.0088 (8)	0.0051 (7)	0.0046 (8)
C5	0.0224 (10)	0.0319 (11)	0.0250 (10)	0.0152 (9)	−0.0013 (8)	0.0039 (8)
C8	0.0125 (8)	0.0162 (8)	0.0234 (9)	0.0038 (7)	0.0010 (7)	0.0061 (7)
C7	0.0127 (8)	0.0168 (9)	0.0230 (9)	0.0042 (7)	0.0029 (7)	0.0061 (7)
C6	0.0177 (9)	0.0201 (9)	0.0355 (11)	0.0091 (8)	0.0042 (8)	0.0093 (8)
C9	0.0162 (9)	0.0159 (9)	0.0215 (9)	0.0037 (7)	−0.0025 (7)	0.0040 (7)
C19	0.0601 (16)	0.0197 (10)	0.0189 (10)	0.0064 (10)	0.0093 (10)	0.0010 (8)
C10	0.0213 (9)	0.0178 (9)	0.0260 (10)	0.0070 (7)	0.0012 (7)	0.0047 (7)
C11	0.0294 (11)	0.0217 (10)	0.0260 (10)	0.0123 (8)	−0.0012 (8)	0.0020 (8)
C12	0.0326 (11)	0.0147 (9)	0.0305 (11)	0.0051 (8)	−0.0091 (9)	0.0019 (8)
C14	0.0208 (9)	0.0193 (9)	0.0221 (9)	0.0029 (8)	−0.0011 (7)	0.0044 (7)
C13	0.0260 (10)	0.0186 (9)	0.0284 (10)	−0.0010 (8)	−0.0035 (8)	0.0085 (8)
C15	0.0210 (9)	0.0131 (8)	0.0194 (9)	0.0017 (7)	−0.0033 (7)	0.0027 (7)
C16	0.0234 (10)	0.0191 (9)	0.0358 (11)	0.0065 (8)	−0.0075 (8)	0.0038 (8)
C17	0.0423 (13)	0.0186 (10)	0.0405 (13)	0.0081 (9)	−0.0213 (11)	0.0045 (9)
C18	0.0674 (17)	0.0159 (10)	0.0187 (10)	0.0045 (10)	−0.0094 (10)	0.0036 (8)

### Geometric parameters (Å, º)

**Table:**

C1—H1A	0.9900	C27—H27	0.9500
C1—H1B	0.9900	C27—C28	1.386 (3)
C1—N1	1.469 (3)	C28—H28	0.9500
C1—P1	1.833 (2)	C29—C30	1.396 (3)
Co1—S1	2.1620 (5)	C29—C34	1.388 (3)
Co1—P1	2.1424 (5)	C30—H30	0.9500
Co1—S2	2.1669 (5)	C30—C31	1.383 (3)
Co1—S3	2.1685 (5)	C31—H31	0.9500
Co1—S4	2.1487 (5)	C31—C32	1.385 (3)
N1—C3	1.462 (3)	C32—H32	0.9500
N1—C5	1.469 (3)	C32—C33	1.382 (3)
Cl1—C35	1.794 (2)	C33—H33	0.9500
S1—C7	1.7282 (18)	C33—C34	1.389 (3)
P1—C2	1.841 (2)	C34—H34	0.9500
P1—C6	1.847 (2)	C35—C35^i^	1.506 (4)
C2—H2A	0.9900	C35—H35A	0.9900
C2—H2B	0.9900	C35—H35B	0.9900
C2—N2	1.472 (3)	C5—H5A	0.9900
S2—C8	1.7192 (19)	C5—H5B	0.9900
N2—C3	1.463 (3)	C8—C7	1.372 (3)
N2—C4	1.460 (3)	C8—C15	1.486 (2)
C3—H3A	0.9900	C7—C9	1.482 (3)
C3—H3B	0.9900	C6—H6A	0.9900
S3—C21	1.7286 (18)	C6—H6B	0.9900
N3—C4	1.468 (3)	C9—C10	1.396 (3)
N3—C5	1.468 (3)	C9—C14	1.405 (3)
N3—C6	1.471 (2)	C19—H19	0.9500
C4—H4A	0.9900	C19—C18	1.377 (4)
C4—H4B	0.9900	C10—H10	0.9500
S4—C22	1.7307 (18)	C10—C11	1.390 (3)
C20—H20	0.9500	C11—H11	0.9500
C20—C19	1.395 (3)	C11—C12	1.386 (3)
C20—C15	1.390 (3)	C12—H12	0.9500
C21—C22	1.365 (3)	C12—C13	1.381 (3)
C21—C29	1.486 (2)	C14—H14	0.9500
C22—C23	1.485 (2)	C14—C13	1.388 (3)
C23—C24	1.390 (3)	C13—H13	0.9500
C23—C28	1.396 (3)	C15—C16	1.395 (3)
C24—H24	0.9500	C16—H16	0.9500
C24—C25	1.386 (3)	C16—C17	1.392 (3)
C25—H25	0.9500	C17—H17	0.9500
C25—C26	1.379 (3)	C17—C18	1.380 (4)
C26—H26	0.9500	C18—H18	0.9500
C26—C27	1.383 (3)		
			
H1A—C1—H1B	108.0	C27—C28—C23	120.67 (19)
N1—C1—H1A	109.4	C27—C28—H28	119.7
N1—C1—H1B	109.4	C30—C29—C21	121.23 (17)
N1—C1—P1	111.30 (13)	C34—C29—C21	119.85 (17)
P1—C1—H1A	109.4	C34—C29—C30	118.91 (17)
P1—C1—H1B	109.4	C29—C30—H30	119.7
S1—Co1—S2	89.729 (19)	C31—C30—C29	120.58 (19)
S1—Co1—S3	88.927 (19)	C31—C30—H30	119.7
P1—Co1—S1	101.94 (2)	C30—C31—H31	119.9
P1—Co1—S2	98.12 (2)	C30—C31—C32	120.1 (2)
P1—Co1—S3	90.975 (19)	C32—C31—H31	119.9
P1—Co1—S4	97.22 (2)	C31—C32—H32	120.2
S2—Co1—S3	170.88 (2)	C33—C32—C31	119.68 (19)
S4—Co1—S1	160.81 (2)	C33—C32—H32	120.2
S4—Co1—S2	88.415 (19)	C32—C33—H33	119.8
S4—Co1—S3	89.895 (19)	C32—C33—C34	120.4 (2)
C3—N1—C1	110.66 (17)	C34—C33—H33	119.8
C3—N1—C5	108.76 (17)	C29—C34—C33	120.29 (19)
C5—N1—C1	111.36 (16)	C29—C34—H34	119.9
C7—S1—Co1	106.05 (7)	C33—C34—H34	119.9
C1—P1—Co1	116.49 (7)	Cl1—C35—H35A	110.0
C1—P1—C2	99.60 (10)	Cl1—C35—H35B	110.0
C1—P1—C6	99.16 (10)	C35^i^—C35—Cl1	108.53 (18)
C2—P1—Co1	119.19 (7)	C35^i^—C35—H35A	110.0
C2—P1—C6	98.56 (10)	C35^i^—C35—H35B	110.0
C6—P1—Co1	119.84 (6)	H35A—C35—H35B	108.4
P1—C2—H2A	109.6	N1—C5—H5A	108.7
P1—C2—H2B	109.6	N1—C5—H5B	108.7
H2A—C2—H2B	108.2	N3—C5—N1	114.18 (16)
N2—C2—P1	110.06 (14)	N3—C5—H5A	108.7
N2—C2—H2A	109.6	N3—C5—H5B	108.7
N2—C2—H2B	109.6	H5A—C5—H5B	107.6
C8—S2—Co1	105.84 (7)	C7—C8—S2	119.61 (14)
C3—N2—C2	111.82 (17)	C7—C8—C15	124.29 (17)
C4—N2—C2	111.77 (17)	C15—C8—S2	115.98 (14)
C4—N2—C3	108.62 (17)	C8—C7—S1	118.77 (14)
N1—C3—N2	114.42 (17)	C8—C7—C9	124.82 (17)
N1—C3—H3A	108.7	C9—C7—S1	116.40 (14)
N1—C3—H3B	108.7	P1—C6—H6A	109.4
N2—C3—H3A	108.7	P1—C6—H6B	109.4
N2—C3—H3B	108.7	N3—C6—P1	111.14 (13)
H3A—C3—H3B	107.6	N3—C6—H6A	109.4
C21—S3—Co1	105.31 (6)	N3—C6—H6B	109.4
C4—N3—C6	110.70 (16)	H6A—C6—H6B	108.0
C5—N3—C4	108.50 (16)	C10—C9—C7	120.40 (17)
C5—N3—C6	111.41 (16)	C10—C9—C14	118.51 (18)
N2—C4—N3	114.23 (16)	C14—C9—C7	121.05 (17)
N2—C4—H4A	108.7	C20—C19—H19	119.8
N2—C4—H4B	108.7	C18—C19—C20	120.4 (2)
N3—C4—H4A	108.7	C18—C19—H19	119.8
N3—C4—H4B	108.7	C9—C10—H10	119.6
H4A—C4—H4B	107.6	C11—C10—C9	120.75 (19)
C22—S4—Co1	105.61 (6)	C11—C10—H10	119.6
C19—C20—H20	119.9	C10—C11—H11	120.0
C15—C20—H20	119.9	C12—C11—C10	120.1 (2)
C15—C20—C19	120.3 (2)	C12—C11—H11	120.0
C22—C21—S3	119.00 (13)	C11—C12—H12	120.1
C22—C21—C29	124.87 (16)	C13—C12—C11	119.86 (19)
C29—C21—S3	116.05 (13)	C13—C12—H12	120.1
C21—C22—S4	119.29 (14)	C9—C14—H14	119.9
C21—C22—C23	125.07 (16)	C13—C14—C9	120.25 (19)
C23—C22—S4	115.62 (13)	C13—C14—H14	119.9
C24—C23—C22	120.60 (17)	C12—C13—C14	120.58 (19)
C24—C23—C28	118.51 (18)	C12—C13—H13	119.7
C28—C23—C22	120.80 (17)	C14—C13—H13	119.7
C23—C24—H24	119.8	C20—C15—C8	121.69 (17)
C25—C24—C23	120.45 (19)	C20—C15—C16	119.03 (18)
C25—C24—H24	119.8	C16—C15—C8	119.29 (18)
C24—C25—H25	119.6	C15—C16—H16	120.0
C26—C25—C24	120.7 (2)	C17—C16—C15	120.1 (2)
C26—C25—H25	119.6	C17—C16—H16	120.0
C25—C26—H26	120.3	C16—C17—H17	119.7
C25—C26—C27	119.41 (19)	C18—C17—C16	120.5 (2)
C27—C26—H26	120.3	C18—C17—H17	119.7
C26—C27—H27	119.9	C19—C18—C17	119.7 (2)
C26—C27—C28	120.24 (19)	C19—C18—H18	120.1
C28—C27—H27	119.9	C17—C18—H18	120.1
C23—C28—H28	119.7		
			
C1—N1—C3—N2	68.0 (2)	C21—C29—C34—C33	178.76 (19)
C1—N1—C5—N3	−67.8 (2)	C22—C21—C29—C30	−108.8 (2)
C1—P1—C2—N2	−49.64 (17)	C22—C21—C29—C34	72.5 (2)
C1—P1—C6—N3	49.67 (16)	C22—C23—C24—C25	−177.3 (2)
Co1—S1—C7—C8	−0.88 (16)	C22—C23—C28—C27	176.84 (19)
Co1—S1—C7—C9	177.96 (12)	C23—C24—C25—C26	0.5 (4)
Co1—P1—C2—N2	−177.43 (12)	C24—C23—C28—C27	0.3 (3)
Co1—P1—C6—N3	177.50 (11)	C24—C25—C26—C27	0.2 (4)
Co1—S2—C8—C7	0.23 (16)	C25—C26—C27—C28	−0.6 (3)
Co1—S2—C8—C15	176.41 (12)	C26—C27—C28—C23	0.4 (3)
Co1—S3—C21—C22	6.17 (16)	C28—C23—C24—C25	−0.7 (3)
Co1—S3—C21—C29	−176.88 (12)	C29—C21—C22—S4	−176.22 (14)
Co1—S4—C22—C21	−6.88 (16)	C29—C21—C22—C23	5.2 (3)
Co1—S4—C22—C23	171.84 (12)	C29—C30—C31—C32	1.4 (3)
N1—C1—P1—Co1	179.98 (12)	C30—C29—C34—C33	0.1 (3)
N1—C1—P1—C2	50.42 (17)	C30—C31—C32—C33	−1.1 (3)
N1—C1—P1—C6	−49.96 (16)	C31—C32—C33—C34	0.2 (3)
S1—C7—C9—C10	41.3 (2)	C32—C33—C34—C29	0.3 (3)
S1—C7—C9—C14	−136.15 (16)	C34—C29—C30—C31	−0.9 (3)
P1—C1—N1—C3	−60.4 (2)	C5—N1—C3—N2	−54.7 (2)
P1—C1—N1—C5	60.7 (2)	C5—N3—C4—N2	55.1 (2)
P1—C2—N2—C3	60.2 (2)	C5—N3—C6—P1	−59.98 (19)
P1—C2—N2—C4	−61.8 (2)	C8—C7—C9—C10	−140.0 (2)
C2—P1—C6—N3	−51.58 (16)	C8—C7—C9—C14	42.6 (3)
C2—N2—C3—N1	−68.7 (2)	C8—C15—C16—C17	−179.60 (18)
C2—N2—C4—N3	68.5 (2)	C7—C8—C15—C20	−123.2 (2)
S2—C8—C7—S1	0.4 (2)	C7—C8—C15—C16	57.0 (3)
S2—C8—C7—C9	−178.29 (14)	C7—C9—C10—C11	−177.73 (18)
S2—C8—C15—C20	60.8 (2)	C7—C9—C14—C13	177.89 (18)
S2—C8—C15—C16	−119.02 (17)	C6—P1—C2—N2	51.23 (17)
C3—N1—C5—N3	54.4 (2)	C6—N3—C4—N2	−67.4 (2)
C3—N2—C4—N3	−55.3 (2)	C6—N3—C5—N1	67.6 (2)
S3—C21—C22—S4	0.4 (2)	C9—C10—C11—C12	−0.2 (3)
S3—C21—C22—C23	−178.16 (14)	C9—C14—C13—C12	−0.1 (3)
S3—C21—C29—C30	74.4 (2)	C19—C20—C15—C8	178.12 (18)
S3—C21—C29—C34	−104.22 (18)	C19—C20—C15—C16	−2.0 (3)
C4—N2—C3—N1	55.1 (2)	C10—C9—C14—C13	0.4 (3)
C4—N3—C5—N1	−54.5 (2)	C10—C11—C12—C13	0.5 (3)
C4—N3—C6—P1	60.85 (19)	C11—C12—C13—C14	−0.3 (3)
S4—C22—C23—C24	48.6 (2)	C14—C9—C10—C11	−0.2 (3)
S4—C22—C23—C28	−127.87 (17)	C15—C20—C19—C18	2.1 (3)
C20—C19—C18—C17	−0.6 (3)	C15—C8—C7—S1	−175.40 (14)
C20—C15—C16—C17	0.5 (3)	C15—C8—C7—C9	5.9 (3)
C21—C22—C23—C24	−132.7 (2)	C15—C16—C17—C18	0.9 (3)
C21—C22—C23—C28	50.8 (3)	C16—C17—C18—C19	−0.9 (3)
C21—C29—C30—C31	−179.60 (18)		

Symmetry code: (i) −*x*, −*y*+1, −*z*+1.

### Hydrogen-bond geometry (Å, º)

**Table:**

*D*—H···*A*	*D*—H	H···*A*	*D*···*A*	*D*—H···*A*
C28—H28···C11^ii^	0.95	2.83	3.575 (3)	136
C19—H19···S4^iii^	0.95	2.78	3.513 (3)	135
C2—H2a···Cl1^i^	0.99	2.88	3.582 (2)	129
C35—H35a···N2	0.99	2.69	3.297 (3)	120
C26—H26···Cl1^iv^	0.95	2.95	3.824 (2)	154

Symmetry codes: (i) −*x*, −*y*+1, −*z*+1; (ii) *x*, *y*+1, *z*; (iii) −*x*+1, −*y*+1, −*z*+1; (iv) −*x*+1, −*y*+2, −*z*+1.
